# Socially Assistive Robots for Community-Dwelling Individuals With Mild Cognitive Impairment and Dementia

**DOI:** 10.1093/geroni/igaf122.4276

**Published:** 2025-12-31

**Authors:** Light Ndurue, Ying-Ling Jao, John Akudugu

**Affiliations:** Pennsylvania State University, University Park, Pennsylvania, United States; Pennsylvania State University, University Park, Pennsylvania, United States; Pennsylvania State University, University Park, Pennsylvania, United States

## Abstract

Mild cognitive impairment (MCI) and dementia are prevalent conditions that significantly impact the quality of life for community-dwelling older adults. Increasing research has focused on the development of socially assistive robots to support this population; yet the evidence has not been systematically synthesized. This integrative review examines research evidence on the effects of socially assistive robots (SARs) for community-dwelling individuals with MCI and dementia. Databases searched included PubMed, CINAHL, and PsycINFO using terms related to MCI, dementia, robotics/assistive technology, and community. Original research that evaluates SARs on community-dwelling individuals with MCI or dementia with reported outcomes were selected, 15 eligible articles identified. Studies reviewed included randomized control trials, quasi-experimental studies, and or pre–post intervention design; with sample size ranging from 30 to 66. The SARs reviewed included zoomorphic and humanoid robots, used either one-to-one interactions or in small group settings. The SARs were designed to provide companionship, prompt conversation, cue reminiscence or music, structure routines, and encourage gentle physical activities. Findings revealed that SARs had positive effects on engagement, pleasure, and social connectedness and demonstrated good usability and acceptability by users. Yet, SARs did not show significant improvement on global cognition or memory. Results on agitation, apathy, and quality of life were mixed across studies. In conclusion, SARs show promising benefits on engagement, positive affect, and social connection for community-dwelling individuals with MCI and dementia. Further research with larger sample sizes and longitudinal designs is needed to establish effective approaches to integrating SARs in clinical practice to support this population.

